# Forebrain-specific deficiency of the GTPase CRAG/Centaurin-γ3 leads to immature dentate gyri and hyperactivity in mice

**DOI:** 10.1016/j.jbc.2021.100620

**Published:** 2021-03-31

**Authors:** Shun Nagashima, Naoki Ito, Reiki Kobayashi, Isshin Shiiba, Hiroki Shimura, Toshifumi Fukuda, Hideo Hagihara, Tsuyoshi Miyakawa, Ryoko Inatome, Shigeru Yanagi

**Affiliations:** 1 Laboratory of Molecular Biochemistry, School of Life Sciences, Tokyo University of Pharmacy and Life Sciences, Hachioji, Tokyo, Japan; 2 Laboratory of Molecular Biochemistry, Department of Life Science, Faculty of Science, Gakushuin University, Toshima-ku, Tokyo, Japan; 3 Division of Systems Medical Science, Institute for Comprehensive Medical Science, Fujita Health University, Toyoake, Aichi, Japan

**Keywords:** CRAG, Centaurin-γ3, AGAP3, immature dentate gyrus, hyperactivity, adult neurogenesis, AP-1, activation protein-1, CRAG, CRMP5-associated GTPase, CRMP, semaphorin response mediator protein, iDG, immature dentate gyrus, PML, promyelocytic leukemia protein, PolyQ, expanded polyglutamine proteins, SRF, serum response factor

## Abstract

Mouse models of various neuropsychiatric disorders, such as schizophrenia, often display an immature dentate gyrus, characterized by increased numbers of immature neurons and neuronal progenitors and a dearth of mature neurons. We previously demonstrated that the CRMP5-associated GTPase (CRAG), a short splice variant of Centaurin-γ3/AGAP3, is highly expressed in the dentate gyrus. CRAG promotes cell survival and antioxidant defense by inducing the activation of serum response factors at promyelocytic leukemia protein bodies, which are nuclear stress-responsive domains, during neuronal development. However, the physiological role of CRAG in neuronal development remains unknown. Here, we analyzed the role of CRAG using dorsal forebrain-specific CRAG/Centaurin-γ3 knockout mice. The mice revealed maturational abnormality of the hippocampal granule cells, including increased doublecortin-positive immature neurons and decreased calbindin-positive mature neurons, a typical phenotype of immature dentate gyri. Furthermore, the mice displayed hyperactivity in the open-field test, a common measure of exploratory behavior, suggesting that these mice may serve as a novel model for neuropsychiatric disorder associated with hyperactivity. Thus, we conclude that CRAG is required for the maturation of neurons in the dentate gyrus, raising the possibility that its deficiency might promote the development of psychiatric disorders in humans.

Abnormalities in brain networks cause psychiatric disorders such as schizophrenia. Studies using knockout (KO) mice are elucidating the relationship between brain structure and behavioral abnormalities. Immature dentate gyrus (iDG) has been reported as a common phenotype in mice with hyperactivity, which is one of the typical behavioral abnormalities (1, 2, 3). In detail, iDG is a phenomenon in which nerve cells (granule cells) of the hippocampal dentate gyrus (DG) are in a pseudo-immature state in terms of molecular and electrophysiological characteristics (4), and similar symptoms have been observed in schizophrenia patients (5), suggesting that iDG is one of the endophenotypes in the brain shared with several neuropsychiatric disorders.

Accumulating reports show that neurogenesis occurs in the hippocampal DG of adults (6, 7, 8). Adult neurogenesis in the DG is approximately divided into six stages: type-1 cells, type-2a cells, type-2b cells, type-3 cells, immature, and mature neurons. Type-1 cells, radial-glia-like neural stem cells, express astrocytic marker glial fibrillary acidic protein (GFAP) and SOX2 and locate in the subgranular zone (SGZ) (9). Type-2a and type-2b cells are classified as intermediate progenitor cells. Type-2a cells express SOX2 and potent capacity of self-renewal. Type-3 cells are neuroblasts that differentiate into immature neurons. Doublecortin (DCX) is expressed in type-2a, type-2b, type-3, and immature neurons. Ca^2+^-binding protein calretinin expression is found in immature neurons, which are more excitable than mature neurons. Mature granule cells express calbindin. Interestingly, the reversal of neuronal maturation is observed in the DG of adult mice (10). Chronic treatment with antidepressant, fluoxetine, causes re-expression of DCX and calretinin in mature granule cells (11). Disruption of maturation and dematuration is associated with iDG.

Neural circuits are complicatedly organized and perform accurate intersynaptic transmission in the networks. Differentiating nerve cells extend axons, reach target cells, and form synapses. The nerve repulsion factors semaphorins play important roles in neural network formation. By screening for signaling targets of semaphorins, we previously identified CRMP5-Associated GTPase (CRAG), a guanosine triphosphatase (GTPase) that is an alternative splicing variant of Centaurin-γ3/AGAP3 (12). Centaurin-γ3, a long isoform of the AGAP3 gene, regulates Arf6-mediated vesicle transport through a Centaurin-γ3-specific region (13), suggesting that CRAG and Centaurin-γ3 have different functions. CRAG is highly expressed in the nervous system. It has been reported that CRAG degrades abnormally expanded polyglutamine (PolyQ), which is a denatured protein, and that lentivirus-mediated CRAG expression in the cerebral brain of polyglutamine disease model mice results in clearance of the PolyQ aggregates and the rescue of ataxia (14). These results suggest the possibility of performing gene therapy for polyglutamine disease using CRAG. In addition, we previously reported that CRAG enhances the cell survival signal against PolyQ accumulation *via* the activation of c-Fos-dependent activator protein-1 (AP-1) (15) and that CRAG activates serum response factor (SRF) by interacting with promyelocytic leukemia protein (PML) through its SUMO-interacting motifs (16). However, the physiological functions of CRAG in the brain remain largely unknown. In the present study, we show that CRAG/Centaurin-γ3-KO mice exhibit hyperactivity and iDG phenotypes. Furthermore, KO of CRAG/Centaurin-γ3 was found to disrupt adult neurogenesis in the DG of the hippocampus. These results suggest that CRAG/Centaurin-γ3 is involved in the pathophysiology of psychiatric disorders.

## Results

**Figure 1:**
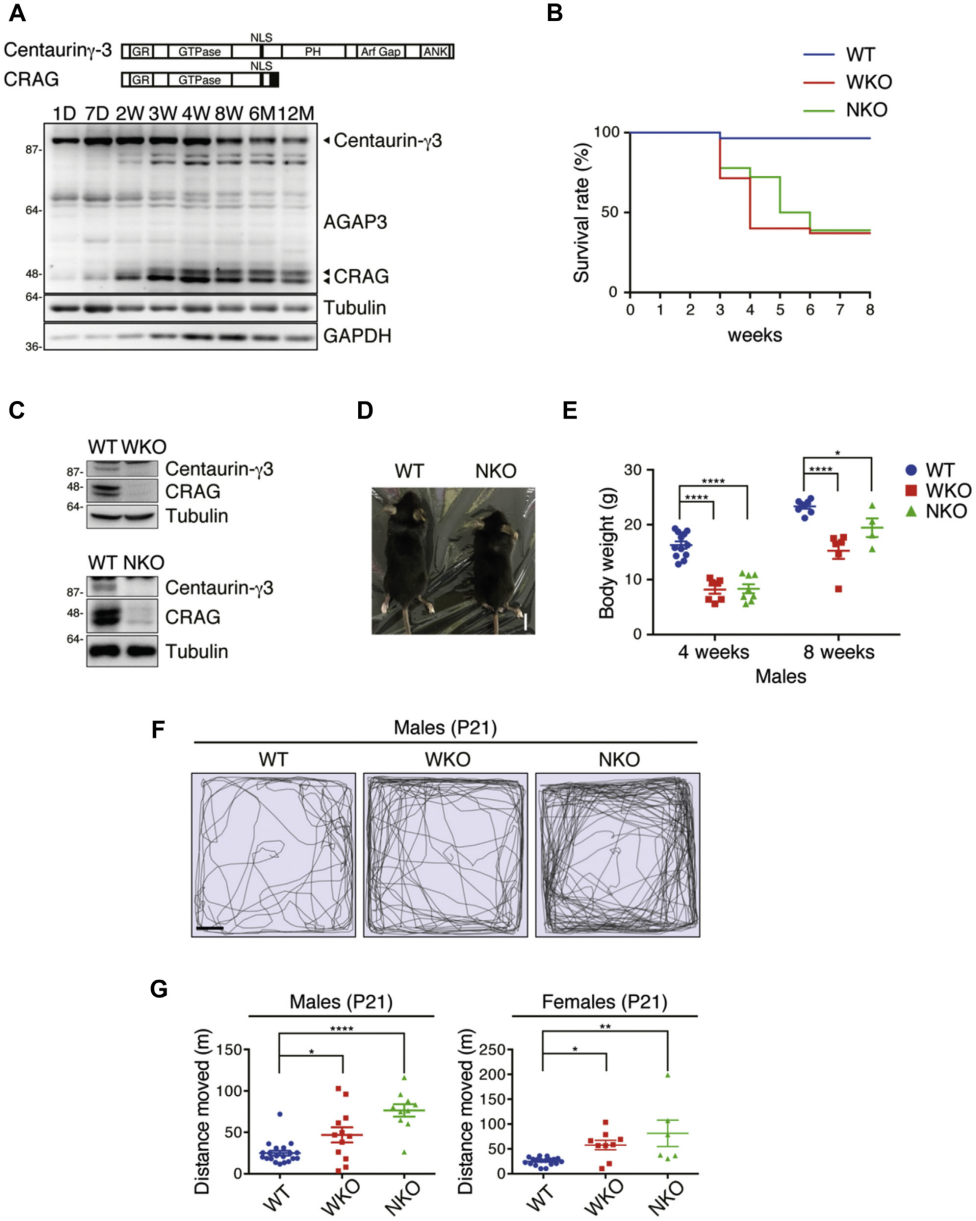
**CRAG/Centaurin-γ3 knockout mice exhibit hyperactive behavior.***A*, time course of Centaurin-γ3 and CRAG expressions in the mouse hippocampi. Immunoblot analysis on whole-cell lysates from the mouse hippocampi at the indicated postnatal stages. Lysates were immunoblotted with anti-AGAP3, anti-α-tubulin, and anti-GAPDH antibodies. *Filled arrowheads* indicate Centaurin-γ3 and CRAG (double bands), respectively. *B*, survival rate of each genotype. (WT, n = 28; WKO, n = 35; NKO, n = 18). *C*, immunoblot analysis indicates the loss of CRAG and Centaurin-γ3 expressions in whole brain of WKO at 8 weeks old and NKO at 14 weeks old. Lysates were immunoblotted with anti-CRAG and anti-α-tubulin antibodies. *D*, NKO mice show smaller body size at 5 weeks old. Scale bar represents 1 cm. *E*, the reduction of body weight in WKO and NKO at the indicated ages. (WT at 4 weeks old, n = 12; WT at 8 weeks old, n = 8; WKO at 4 weeks old, n = 7; WKO at 8 weeks old, n = 6; NKO at 4 weeks old, n = 8; NKO at 8 weeks old, n = 4). *Dot plots* represent mean ± SEM. Data were analyzed with the two-way ANOVA, Sidak's multiple comparisons test. ∗*p* < 0.05, ∗∗∗∗*p* < 0.0001. *F* and *G*, increased locomotor activity of WKO and NKO at postnatal day (P) 21 in the open-field test. Tracing of mouse movement (*F*) and total distance moved (*G*) during the 10-min test period. (Male WT, n = 20; Male WKO, n = 12; Male NKO, n = 10; Female WT, n = 18; Female WKO, n = 9; Female NKO, n = 6). *Dot plots* represent mean ± SEM. Data were analyzed with unpaired *t*-test with equal SD. ∗*p* < 0.05, ∗∗*p* < 0.01, ∗∗∗∗*p* < 0.0001. D, days; M, months; W, weeks.

CRAG, a splicing variant of Centaurin-γ3, is dominantly expressed in the brain. Western blot analysis of hippocampal lysates showed that CRAG expression was gradually enhanced from 2-week-old mice (Fig. 1*A*). We previously generated whole-body CRAG/Centaurin-γ3-KO mice (WKO), which may selectively reflect CRAG dysfunction (16). Overall, 60% of WKO mice died within 2 months of birth (Fig. 1*B*). To determine whether the cause of death was neuronal dysfunction, we generated central nervous system (CNS)-specific CRAG/Centaurin-γ3-KO (NKO) mice by crossing CRAG/Centaurin-γ3 floxed mice with nestin-Cre transgenic mice (17). Western blot analysis showed that the bands of CRAG and Centaurin-γ3 disappeared in the whole brain lysates of WKO and NKO (Fig. 1*C*). The NKO mice had survival rates similar to those of WKO mice (Fig. 1*B*), indicating that the WKO mice had died from CNS dysfunction. WKO and NKO had smaller body size and weight than wild-type (WT) mice (Fig. 1, *D* and *E*). To further assess the CRAG/Centaurin-γ3 KO mice, we performed the open-field test. Both WKO and NKO exhibited hyperactivity and increased total distance traveled (Fig. 1, *F* and *G*). Taking these findings together, CRAG/Centaurin-γ3 loss in the CNS causes impairment of body growth and abnormal behavior.

**Figure 2:**
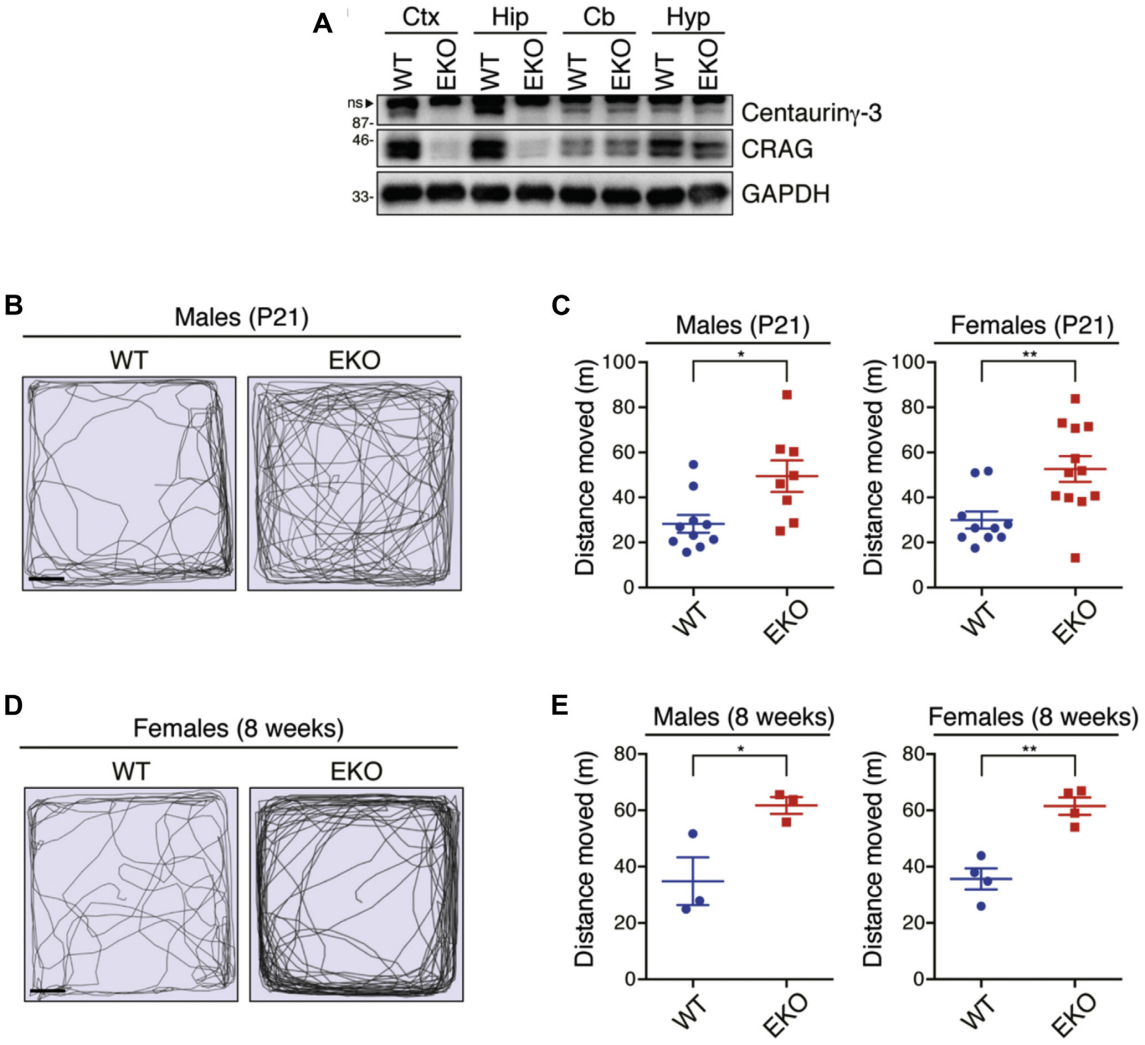
**Emx1-Cre-mediated CRAG/Centaurin-γ3 knockout mice show hyperactivity.***A*, immunoblot analysis indicates the loss of Centaurin-γ3 and CRAG expressions in the cerebral cortex and hippocampus but not cerebellum and hypothalamus of EKO. *B*–*E*, increased locomotor activity of EKO at P21 and 8 weeks old in the open-field test. Tracing of mouse movement (*B* and *D*) and total distance moved (*C* and *E*) during the 10-min test period at P21 and 8 weeks old, respectively. (Male WT at P21, n = 10; Male EKO at P21, n = 8; Female WT at P21, n = 10; Female EKO at P21, n = 12; Male WT at 8 weeks old, n = 3; Male EKO at 8 weeks old, n = 3; Female WT at 8 weeks old, n = 4; Female EKO at 8 weeks old, n = 4). *Dot plots* represent mean ± SEM. Data were analyzed with the ordinary one-way ANOVA, Tukey's multiple comparisons test. ∗*p* < 0.05, ∗∗*p* < 0.01. Cb, Cerebellum; Ctx, Cerebral cortex; Hip, Hippocampus; Hyp, Hypothalamus; ns, non-specific bands.

Mice with the abnormalities of hippocampal neurons exhibit hyperactivity (3). To test this possibility, we generated dorsal forebrain-specific CRAG/Centaurin-γ3-KO (EKO) mice by crossing CRAG/Centaurin-γ3 floxed mice with Emx1-Cre transgenic mice (18). Because Emx1-Cre drives Cre expression mainly in the cerebral cortex and hippocampus, the deletion of CRAG and Centaurin-γ3 was restricted in the cerebral cortex and hippocampus (Fig. 2*A*). As expected, EKO was associated with increased locomotor activity in mice at both P21 and 8 weeks old (Fig. 2, *B*–*E*). The open-field test is used to assess anxiety behavior (19). An increased time spent in the center area suggests less anxiety. To investigate whether EKO displays increased anxiety-like behavior, we analyzed the time spent in the center area (Fig. S1). Any significant difference was not observed in the time spent in the center area, suggesting that hyperactivity in EKO was not caused by anxiety. These findings suggest that forebrain-specific deletion of CRAG/Centaurin-γ3 causes hyperactivity in mice.

**Figure 3:**
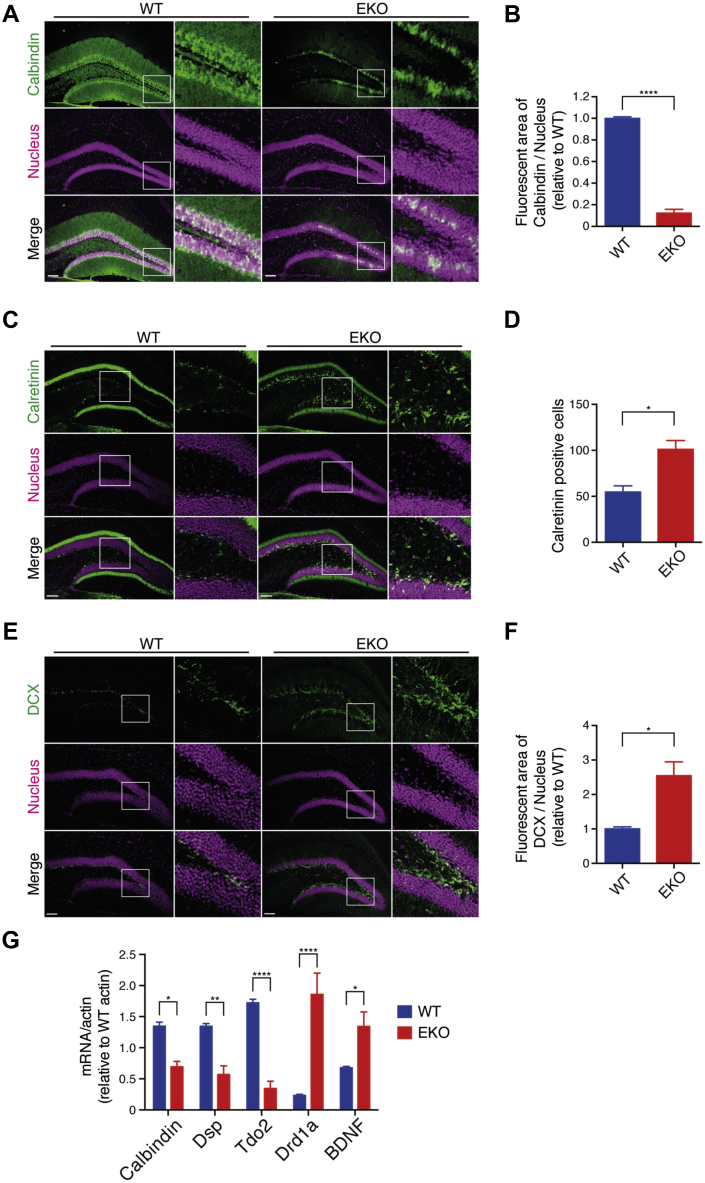
**Emx1-Cre-mediated CRAG/Centaurin-γ3 knockout mice exhibit immature dentate gyrus.***A* and *B*, reduced mature neurons in the DG of EKO. Representative images of WT and EKO at 8 weeks old with calbindin and Hoechst 33258 (Nucleus) labeled (*A*). Scale bars, 100 μm. Quantification of calbindin-positive area in the region of interest (ROI) of 8-week-old mice (*B*). Data represent mean ± SEM (n = 3). Data were analyzed with unpaired *t*-test with equal SD. ∗∗∗∗*p* < 0.0001. *C*–*F*, increased immature neurons in the DG of EKO. Representative images of WT and EKO at the 10 weeks old with Hoechst 33258 (Nucleus) and Calretinin (*C*), and DCX (*E*) labeled. Scale bars, 100 μm. Quantification of calretinin-positive cells (*D*) and DCX-positive area (*F*) in the ROI of 8- to 10-week-old mice. Data represent mean ± SEM (n = 3). Data were analyzed with unpaired *t*-test with equal SD. ∗*p* < 0.05. *G*, gene expression of iDG markers. Quantification of iDG markers mRNA levels in the 8-week-old mice hippocampi by qRT-PCR normalized to ß-actin mRNA. Data represent mean ± SEM (n = 6). Data were analyzed with the two-way ANOVA, Sidak's multiple comparisons test. ∗*p* < 0.05, ∗∗*p* < 0.01, ∗∗∗∗*p* < 0.0001. BDNF, Brain-derived neurotrophic factor; Drd1a, Dopamine receptor D1A; Dsp, Desmoplakin; Tdo2, Tryptophan 2,3-dioxygenase.

A reduction in mature neuronal markers and an increase in immature neuronal markers are observed in the hippocampus of mice showing increased locomotor activity (3, 20). To test mature neurons in the DG, we performed immunostaining with anti-Calbindin antibody. The Calbindin-positive area was significantly reduced in the DG of EKO (Fig. 3, *A* and *B*). The phenotype of mature neuron reduction in EKO may not have been due to apparent cell loss, since nuclear staining was observed uniformly throughout the DG granule cell layer. We checked the calbindin expression in the cerebral cortex (Fig. S2*A*) and the hippocampal CA1 regions (Fig. S2*B*). There was no significant difference in the calbindin expression level in the cerebral cortex of EKO. Although calbindin-positive cells were not significantly reduced in CA1 of EKO, the signal intensity of calbindin was reduced. Compared with the DG, the differences of the calbindin expression are much less in these regions. To characterize the decreased neurons in the DG of EKO, we performed the histological analysis with neuronal cell marker NeuN. NeuN-positive cells were not altered in the DG of EKO (Fig. S2*C*), indicating that the calbindin-positive mature neurons are selectively reduced in the DG of EKO. NeuN-positive cells include both calretinin-positive cells and immature neurons (21, 22). As expected, calretinin-positive neurons were increased in the DG of EKO (Fig. 3, *C* and *D*). In addition, DCX, which is expressed in neuronal progenitors, neuroblasts, and immature neurons, was significantly increased in the DG of EKO (Fig. 3, *E* and *F*). Histological analysis of EKO at P28 showed that calbindin-positive cell layers in the DG of EKO were shorten as compared with WT (Fig. S3*A*). Calbindin-positive area was reduced in the DG of EKO at P28 (Fig. S3*B*) as well as in the DG of 8-week-old EKO. On the other hand, no significant difference in DCX-positive area was observed between WT and EKO at P28 (Fig. S3, *A* and *C*). Many DCX-positive cells in both WT and EKO suggest that neurogenesis is active at this stage. It is therefore considered that no significant difference was observed in DCX-positive cells at P28 due to active neurogenesis. Furthermore, we evaluated molecular markers for iDG using qRT-PCR (23). Consistent with the immunohistochemical analysis, Calbindin mRNA levels showed significant downregulation in the hippocampus of EKO (Fig. 3*G*). Desmoplakin (Dsp) and tryptophan 2,3-dioxygenase (Tdo2) are maturation marker genes that were highly expressed in the DG and were significantly reduced in the hippocampus of EKO. We additionally observed increased mRNA levels of iDG markers such as dopamine receptor D1A (Drd1a) and brain-derived neurotrophic factor (BDNF). Taking these findings together, EKO displays iDG phenotype in terms of molecular expression patterns.

**Figure 4:**
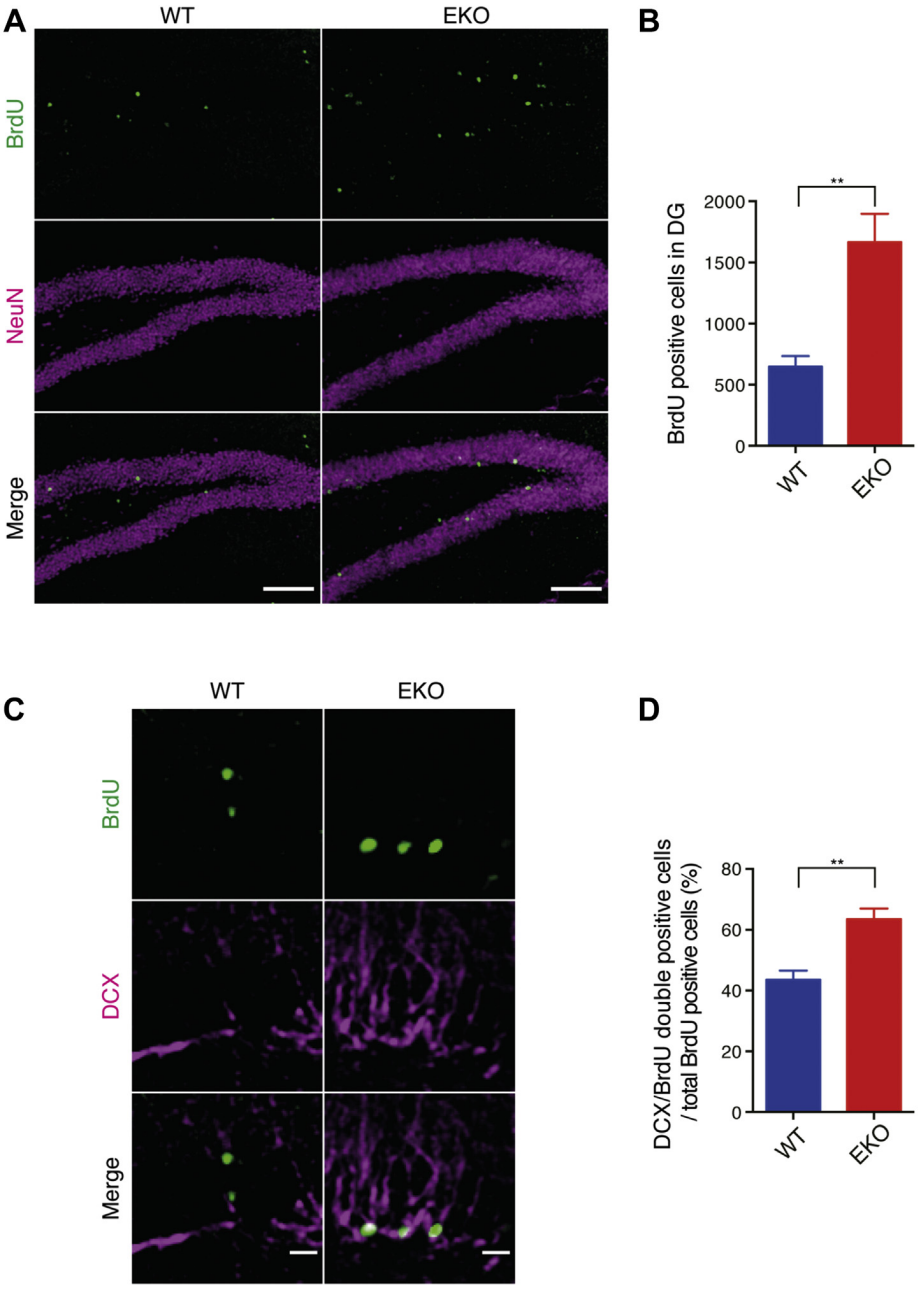
**Loss of CRAG/Centaurin-γ3 enhances adult neurogenesis in the dentate gyrus.***A* and *B*, BrdU-positive cells are increased in the DG of EKO. Mice were analyzed 28 days after the last BrdU injection. Representative images of WT and EKO at 12 weeks old with BrdU and NeuN labeled (*A*). Scale bars, 100 μm. Quantification of BrdU-positive cells in the DG of 12-week-old mice (*B*). *C* and *D*, CRAG loss affects neuronal cell maturation. Mice were analyzed 28 days after the last BrdU injection. Representative images of WT and EKO at 12 weeks old with BrdU and DCX labeled (*C*). Scale bars, 20 μm. Quantification of DCX-positive BrdU labeling cells in total BrdU labeling cells (*D*). Data represent mean ± SEM (n = 4). Data were analyzed with unpaired *t*-test with equal SD. ∗∗*p* < 0.01.

To demonstrate how the loss of CRAG/Centaurin-γ3 contributes to the maturational abnormality of the hippocampal granule cells, we analyzed neurogenesis in the DG because abnormally generated neurons affect hippocampal maturation and locomotor activity (20). To assess the level of adult neurogenesis in the DG, we examined the number of 5-bromo-2'-deoxyuridine (BrdU)-positive cells after the intraperitoneal injection of BrdU. BrdU-positive cells were significantly increased in the DG of EKO compared with the level in WT (Fig. 4, *A* and *B*), indicating enhanced neurogenesis in the DG of EKO. We traced the BrdU-incorporated cells in the DG. The population of DCX-positive BrdU-labeled cells was increased in the DG of EKO (Fig. 4, *C* and *D*). To understand the role of CRAG/Centaurin-γ3 in adult neural stem cell differentiation, we traced the BrdU-incorporated cells and colabeled with SOX2, a neuronal progenitor cell marker, 1 day after BrdU injections (Fig. S4*A*). Consistent with Figure 4*B*, BrdU-positive cells were increased in the DG (Fig. S4*B*). SOX2-positive BrdU-labeled cells were increased, whereas SOX2-negative BrdU-labeled cells were not altered (Fig. S4*C*). SOX2-negative BrdU-labeled cells include neuroblasts, immature neurons, and mature neurons, indicating that newly generated cells can differentiate into neuroblasts and neurons in the DG of EKO. These results suggest that CRAG/Centaurin-γ3 ablation causes the accumulation of immature neurons. To examine whether CRAG/Centaurin-γ3 loss affects neuronal maturation at embryonic development stage, histological analysis was performed at P10 after BrdU injection at E15 (Fig. S5*A*). The number of BrdU-positive cells was not changed, suggesting that CRAG/Centaurin-γ3 did not affect embryonic neurogenesis (Fig. S5*B*). Moreover, BrdU-positive cells were observed in the granule cell layer, which is apart from the SGZ where neurogenesis occurs. This suggests that CRAG/Centaurin-γ3 is not critical for neuronal maturation at embryonic development stage.

## Discussion

Neurogenesis, which is important for the formation of neural networks, is most activated during fetal life and decreases with growth, but it was found that adult neurogenesis occurs in certain areas of the olfactory bulb and hippocampus of the adult brain. Dysregulation of adult neurogenesis in the DG is associated with psychiatric disorders and abnormal behaviors (24). BMP/RA-induced neural-specific protein-1 (BRINP1) suppresses the cell cycle in neural stem cells (25). In addition, BRINP1 KO mice exhibit hyperactivity accompanied by an increase of hippocampal neurogenesis (20). Moreover, increasing adult neurogenesis by Bax deletion enhances exploratory behavior but not locomotor activity in the home cage (26). These studies prompted us to examine whether CRAG deletion also causes a similar increase of adult neurogenesis because CRAG overexpression in cells induces growth arrest through the functional modification of PML body. In our previous study, CRAG was shown to bind with PML *via* its SUMO-interacting motifs (16). PML, a tumor suppressor, regulates cell-cycle progression of the nervous system as PML loss was found to disrupt neuronal cell differentiation and increase neurogenesis (27). Interestingly, CRAG expression is gradually increased in the hippocampus from 2 weeks old (Fig. 1*A*), suggesting that CRAG affects adult neurogenesis in the DG but not neurogenesis during the fetal stage. The cyclin-dependent kinase inhibitor P57KIP2 regulates both embryonic and adult neurogenesis because its deletion was found to promote neurogenesis (28, 29). Although embryonic neurogenesis and adult neurogenesis have a common regulatory mechanism, it is speculated that they have some differences in terms of the regulatory machinery. CRAG could be involved in an adult neurogenesis-specific regulatory system, rather than being involved in the embryonic period.

CRAG/Centaurin-γ3 mice showed iDG phenotypes. It has been reported that chronic treatment with the antidepressant fluoxetine, a selective serotonin reuptake inhibitor, induces the reversal of neuronal maturation in the DG and causes iDG phenotypes in adult mice (11, 30). The reversal of neuronal maturation in adult mice could also be induced by pilocarpine-induced seizures (23) and electroconvulsive stimulations (31). Given that the effects of CRAG deficiency appear after 2 weeks of age (Fig. 1*A*), the decreased calbindin-immunoreactive area in CRAG/Centaurin-γ3 KO mice may be due in part to the reversal of neuronal maturation. Moreover, deletion of CRAG/Centaurin-γ3 disrupted neuronal maturation in the DG (Fig. 4, *C* and *D*), indicating that CRAG/Centaurin-γ3 is involved in neuronal maturation. We previously demonstrated that CRAG activates transcription factors such as SRF and AP-1 (16). SRF is highly expressed in the granular cells of the DG (32), regulates neurite outgrowth (33), and contributes to hippocampal layer and nerve fiber organization (34). These reports indicate that SRF is an important regulator of hippocampal development. In addition, forebrain-specific SRF KO mice exhibit increased locomotor activity, as do CRAG/Centaurin-γ3 KO mice (35). Downregulation of SRF in CRAG/Centaurin-γ3 KO mice may suppress neuronal maturation and thereby induce iDG phenotypes.

We previously demonstrated that lentivector-mediated expression of CRAG in Purkinje cells of PolyQ disease model mice extensively cleared PolyQ aggregates and reactivated dendritic differentiation, resulting in a striking rescue from ataxia (14). The phenotype of decreased calbindin-positive mature neurons in the hippocampus is observed in mouse models of neurodegenerative disease such as dementia with Lewy bodies (4) and Alzheimer’s disease (36), suggesting that the phenotype of iDG can be an intermediate phenotype between psychiatric and neurodegenerative diseases. CRAG gene delivery or drugs that can activate CRAG in the brain may also have therapeutic potential in the treatment of mental illness.

## Experimental procedures

### Animal

All animals were maintained under university guidelines for the care and use of animals. The experiments were performed after securing Tokyo University of Pharmacy and Life Sciences Animal Use Committee Protocol approval. CRAG flox/flox mice (Accession No. CDB0630K: http://www2.clst.riken.jp/arg/mutant%20mice%20list.html) and WKO mice were reported previously (16). Nestin-Cre mice and Emx1-Cre mice were obtained from the Jackson Laboratory and the RIKEN BRC, respectively (17, 18). Cre-negative CRAG flox/flox and CRAG +/flox mice were used as WT mice. Genotype was confirmed by tail tipping mice at around 1 month. Mice were genotyped for CRAG gene using PCR primer A, 5’-CTCAGGATGACTCCCGAACTCTATACGG-3’, and primer B, 5’-CTGGCAGGGCCTGGTAGATGTGCTTCATTG-3’, primer C, 5’-GATTGGCGGCACTGATGGCATCTGTTGG-3’. Mice were genotyped for Cre gene using PCR primer, 5’-GTTTCACTGGTTATGCGGCGG-3’, and primer, 5’-TTCCAGGGCGCGAGTTGATAG-3’. Mice were genotyped for IL-2 gene as an internal control using PCR primer, 5’-CTAGGCCACAGAATTGAAAGATCT-3’, and primer, 5’-GTAGGTGGAAATTCTAGCATCATCC-3’. Body weight was measured at 4-week-old and 8-week-old mice.

### Antibodies

Rabbit polyclonal anti-CRAG antibody was described previously (12). Rabbit polyclonal anti-AGAP3 (NBP1-22968) antibody was purchased from Novus Biologicals. Mouse monoclonal anti-α-tubulin (T9026) and anti-Calretinin (C7479) antibodies were purchased from Sigma. Mouse monoclonal anti-GAPDH (ACR001P) antibody was purchased from Origene. Mouse monoclonal anti-Calbindin D-28K (300) antibody was purchased from Swant. Mouse monoclonal anti-NeuN (MAB377) antibody was purchased from Merck Millipore. Rabbit polyclonal anti-Doublecortin (ab77450) antibody was purchased from Abcam. Rat monoclonal anti-BrdU (MCA2060) antibody was purchased from AbD Serotec.

### Western blot analysis

For the brain samples, the brain was resuspended in lysis buffer (10 mM Tris-HCl (pH7.4), 150 mM NaCl, 5 mM EDTA, 1% Triton X-100, 0.1% SDS, 0.05% DOC, 10 μg/ml Aprotinin, 10 μg/ml Leupeptin, 1 mM PMSF). The insoluble debris was removed by centrifugation at 15,000*g* for 15 min at 4 °C. Samples were mixed with 2x SDS-PAGE sample buffer (125 mM Tris-HCl (pH6.8), 4% SDS, 10% sucrose, 10% 2-mercaptoethanol, 0.01% bromophenol blue). The protein concentrations were measured by Protein Quantification Assay (TaKaRa). Samples were diluted to 0.15 μg/μl with 1x SDS-PAGE sample buffer and then incubated at 95 °C for 5 min. Samples were separated by SDS-PAGE and then transferred to PVDF membranes (Millipore). For western blot analysis, the membranes were blocked with 2.5% skim milk in Tris-buffered saline with 0.1% Tween-20 (TBST). Membranes were incubated with first antibody at 4 °C overnight and second antibody for 1 h at room temperature. The protein bands were treated with enhanced chemiluminescence reagent (Millipore) and detected by using LuminoGraph (ATTO).

### Histology and immunohistochemistry

Mice were perfused with 4% paraformaldehyde (PFA) and the brains were isolated. Brains were fixed in 4% PFA overnight at 4 °C, and 40 μm slice sections were prepared. For fluorescent stains, sections were incubated in 0.1% Triton X-100/PBS for 15 min, washed with PBS, blocked with 0.5% blocking reagent (Roche) in 0.1% Triton X-100/PBS, and incubated for two nights at 4 °C with primary antibodies. Slices were incubated overnight with Alexa-conjugated secondary antibodies and counterstained with Hoechst 33258. For BrdU staining, sections were incubated in 0.4% Triton X-100/PBS for 10 min, washed with PBS, treated with 2 M HCl for 25 min at 37 °C, neutralized with 40 mM boric acid, washed with PBS, blocked with 0.5% blocking reagent (Roche) in 0.1% Triton X-100/PBS, and incubated for two nights at 4 °C with primary antibodies. Slices were incubated overnight with Alexa-conjugated secondary antibodies. The samples were analyzed using an Olympus FV1000-D confocal fluorescence microscope. The fluorescent area was manually measured in the ROI (0.0625 mm^2^) of the DG using Fiji software (37). Calretinin positive cells were manually counted in the ROI (0.24 mm^2^) of the DG.

### BrdU injections

For BrdU labeling, 8-week-old mice received five consecutive daily injections of BrdU (100 mg⁄kg body weight per day) intraperitoneally, and mice were killed 4 weeks and 1 day following the last BrdU injection, respectively. BrdU (100 mg/kg body weight) was injected intraperitoneally into mice at E15 and the mice were analyzed at P10.

### Quantification of BrdU-positive cells

For the stereological analysis of the total BrdU-positive cells at 8 weeks old and 12 weeks old, every sixth 40-μm-thick section was counted throughout the hippocampus, and the sum was multiplied by 6 to provide an estimate of the total number of BrdU-positive cells in the entire region. For the analysis of the BrdU-positive cells at P10, the number of BrdU-positive cells was the average number of BrdU-positive cells in the DG of two sections.

### RNA isolation and quantitative polymerase chain reaction (qPCR)

Total RNA was obtained from the hippocampus of 8-week-old mice. RNA isolation and qPCR were performed as previously described (4).

### Open-field test

Locomotor activity was measured using an open-field test (60 × 60 × 30 cm). Mice were placed in the center of apparatus, and mouse movements were recorded with a video camera for 10 min. Total distance traveled was measured using Meander. The center area was defined as the central 30 × 30 cm portion.

### Statistical analyses

All statistical analyses were performed using PRISM 6 (Graph Pad). ∗*p* < 0.05, ∗∗*p* < 0.01, ∗∗∗*p* < 0.001 and ∗∗∗∗*p* < 0.0001 were considered significant.

## Data availability

The data in the current study are available from the corresponding author on reasonable request.

## Supporting information

This article contains supporting information.

## Conflict of interest

The authors declare that they have no conflict of interest.
