# Supplementary material for: Forebrain-specific deficiency of the GTPase CRAG/Centaurin-γ3 leads to immature dentate gyri and hyperactivity in mice
Source: J Biol Chem. 2021 Mar 31;296:100620. doi: 10.1016/j.jbc.2021.100620 (PMC8099661; doi:10.1016/j.jbc.2021.100620)
Supplement: Supplemental Figures S1–S5 [file mmc1.pdf]

**SupportingInformation**

# Immature dentate gyrus and abnormal behavior of CRAG/Centaurin-γ3 knockout mice

Shun Nagashima[1]*, Naoki Ito[1, 2], Reiki Kobayashi[1], Isshin Shiiba[1, 2], Hiroki Shimura[1], Toshifumi Fukuda[1], Hideo Hagihara[3], Tsuyoshi Miyakawa[3], Ryoko Inatome[2], Shigeru Yanagi[1,2]*

[1] Laboratory of Molecular Biochemistry, School of Life Sciences, Tokyo University of Pharmacy and Life Sciences, Hachioji, Tokyo,192-0392, Japan

[2] Laboratory of Molecular Biochemistry, Department of Life Science, Faculty of Science, Gakushuin University, 1-5-1 Mejiro, Toshima-ku, Tokyo, 171-8588, Japan

[3] Division of Systems Medical Science, Institute for Comprehensive Medical Science, Fujita Health University, Toyoake, Aichi, 470-1192, Japan

*Corresponding author: Shigeru Yanagi and Shun Nagashima

E-mail: syanagi@ls.toyaku.ac.jp and nagashi@toyaku.ac.jp

**Running title:** CRAG/Centaurin-γ3 loss causes immature dentate gyrus

**Keywords:** CRAG, Centaurin-γ3, AGAP3, immature dentate gyrus, hyperactivity, adult neurogenesis

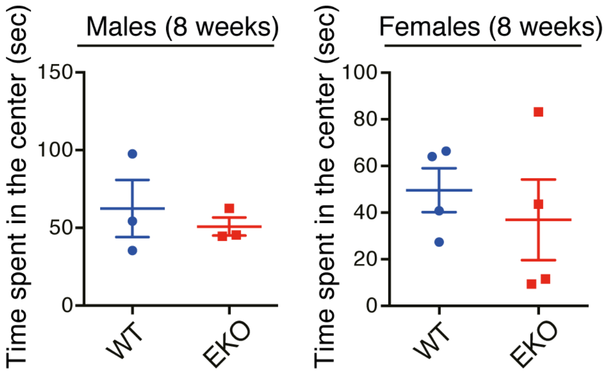


**Fig. S1.** Emx1-Cre mediated CRAG/Centaurin-γ3 knockout mice did not display anxiety behavior.

No significant difference between WT and EKO on time spent in the center area in the open-field test. (Male WT at 8 weeks old, n = 3; Male EKO at 8 weeks old, n = 3; Female WT at 8 weeks old, n = 4; Female EKO at 8 weeks old, n = 4). Dot plots represent mean ± SEM. Data were analyzed with Unpaired t-test with equal SD.

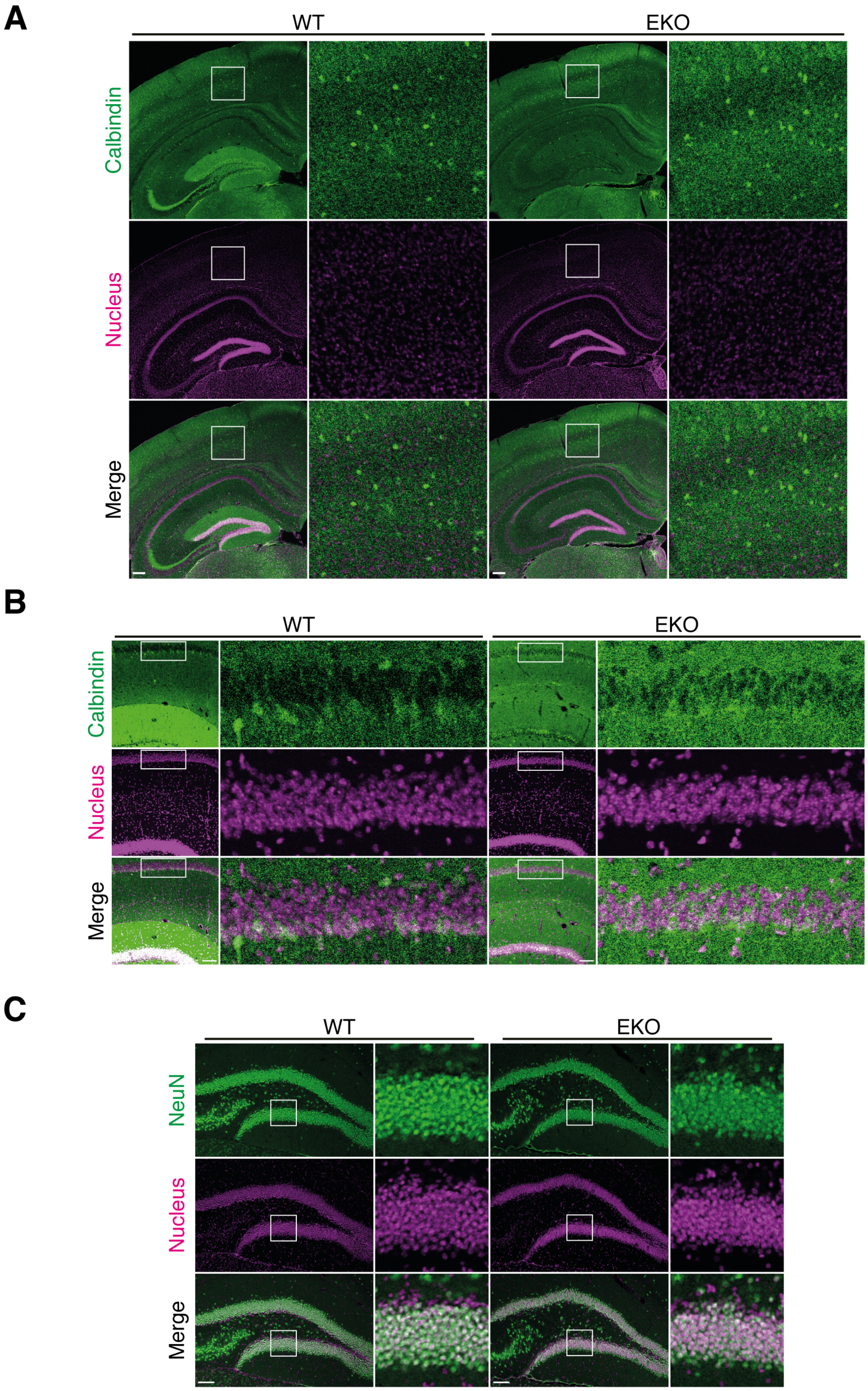
A
WT
EKO
Calbindin
Nucleus
Merge
B
WT
EKO
Calbindin
Nucleus
Merge
C
WT
EKO
NeuN
Nucleus
Merge

**Fig. S2.** Calbindin-positive neurons were specifically reduced in the dentate gyrus of EKO.
(A, B) Calbindin-positive cells were not significantly changed in the cerebral cortex (A) and CA1 (B). Representative images of WT and EKO at 10 weeks old with calbindin and Hoechst 33258 (Nucleus) labeled. Scale bars indicate 200 μm (A) and 100 μm (B), respectively.
(C) NeuN-positive cells were not altered in the DG of EKO. Representative images of WT and EKO at 10 weeks old with NeuN and Hoechst 33258 (Nucleus) labeled. Scale bars, 100 μm.

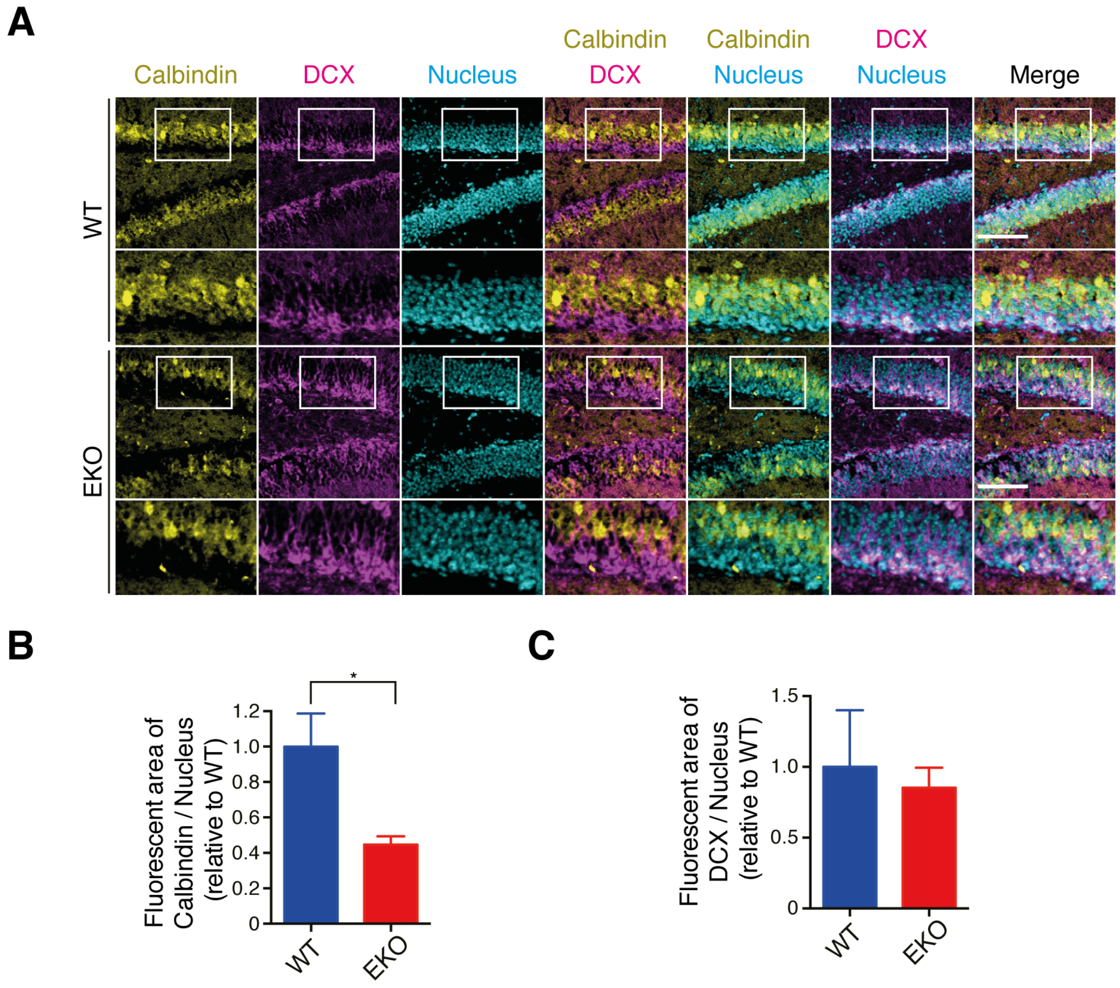


**Fig. S3.** CRAG/Centaurin-γ3 loss reduces calbindin-positive cells neurons in the dentate gyrus at P28.

(A) Representative images of WT and EKO at P28 with calbindin, DCX and Hoechst 33258 (Nucleus) labeled. Scale bars, 100 μm.

(B, C) Quantification of calbindin-positive (B) DCX-positive (C) area in the ROI. Data represent mean ± SEM (n = 3). Data were analyzed with Unpaired t-test with equal SD. *P < 0.05.

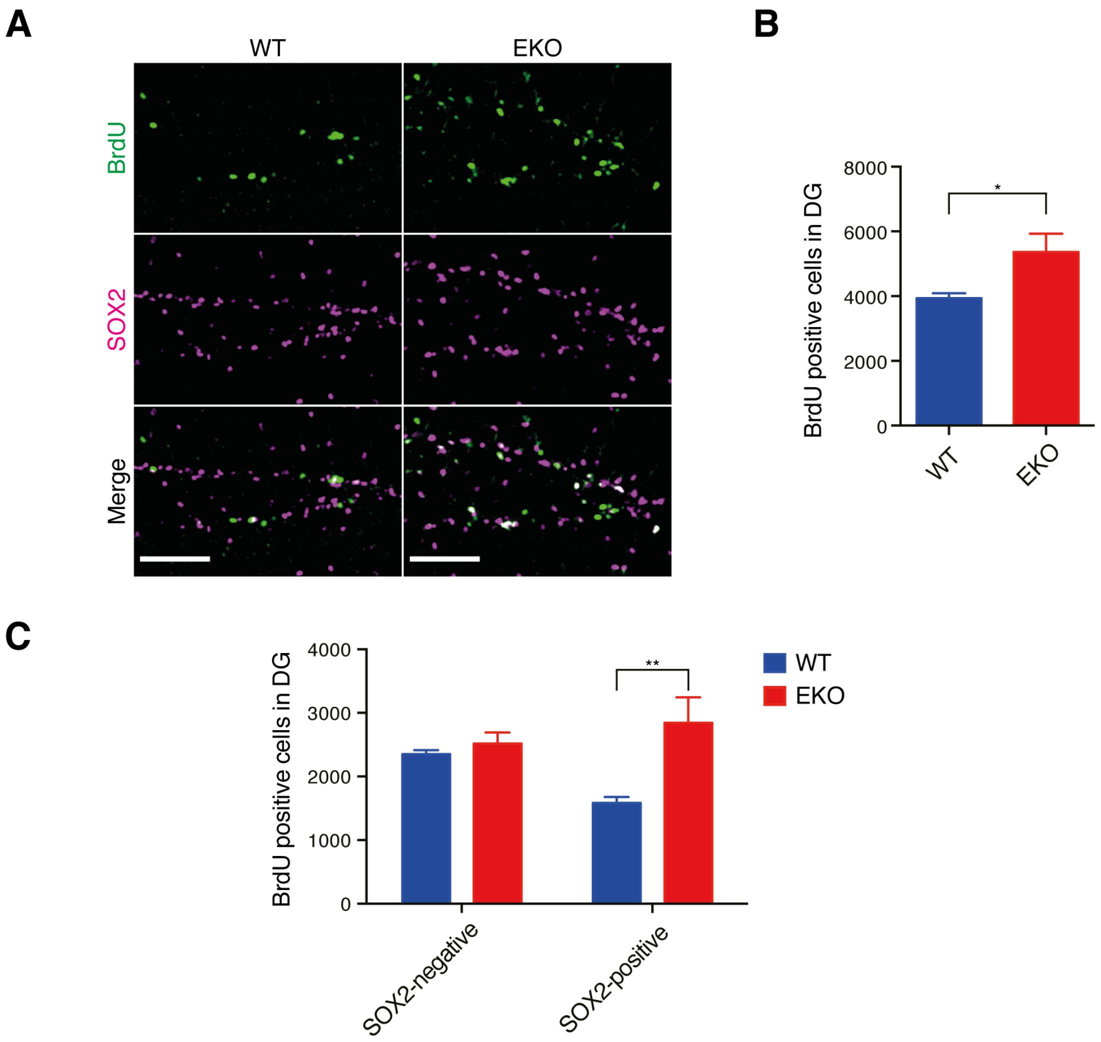


**Fig. S4.** CRAG/Centaurin-γ3 did not affect embryonic neurogenesis and neuronal maturation. (A-C) Mice were analyzed 1 day after the last BrdU injection. Representative images of WT and EKO at 8 weeks old with BrdU and SOX2 labeled (A). Scale bars, 100 μm. Quantification of BrdU-positive cells in the DG of 8 weeks old mice (B). Quantification of SOX2-positive and SOX2-negative BrdU labeling cells (C). Data represent mean ± SEM (n = 5). Data were analyzed with Unpaired t-test with equal SD (B) and the two-way ANOVA, Sidak's multiple comparisons test (C), respectively. *P < 0.05. **P < 0.01.

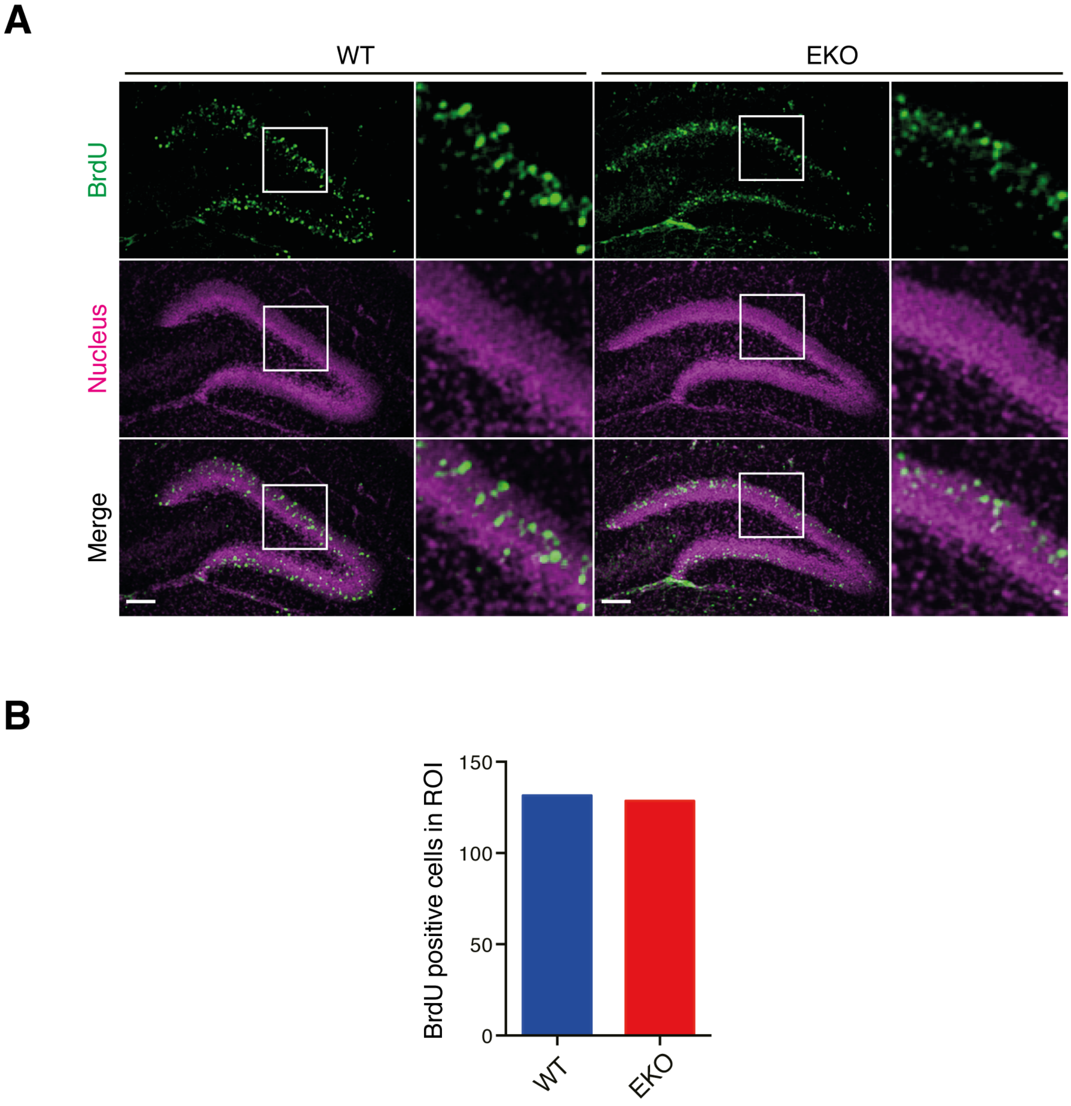


**Fig. S5.** Loss of CRAG/Centaurin-γ3 did not affect embryonic neurogenesis and neuronal maturation.

(A) BrdU-positive cells were not changed in the DG of EKO. Mice were injected with BrdU at E15 and analyzed at P10. Representative images of WT and EKO at P10 with BrdU and Hoechst 33258 (Nucleus) labeled. Scale bars, 100 µm.

(B) Quantification of BrdU-positive cells in the DG of P10 mice (n = 1).
